# Item parameters dissociate between expectation formats: a regression analysis of time-frequency decomposed EEG data

**DOI:** 10.3389/fpsyg.2014.00847

**Published:** 2014-08-12

**Authors:** Irene F. Monsalve, Alejandro Pérez, Nicola Molinaro

**Affiliations:** ^1^BCBL. Basque Center on Cognition, Brain and LanguageDonostia, Spain; ^2^Ikerbasque, Basque Foundation for ScienceBilbao, Spain

**Keywords:** neuronal oscillations, gamma, theta, anticipatory processes, reading

## Abstract

During language comprehension, semantic contextual information is used to generate expectations about upcoming items. This has been commonly studied through the N400 event-related potential (ERP), as a measure of facilitated lexical retrieval. However, the associative relationships in multi-word expressions (MWE) may enable the generation of a categorical expectation, leading to lexical retrieval *before* target word onset. Processing of the target word would thus reflect a target-identification mechanism, possibly indexed by a P3 ERP component. However, given their time overlap (200–500 ms post-stimulus onset), differentiating between N400/P3 ERP responses (averaged over multiple linguistically variable trials) is problematic. In the present study, we analyzed EEG data from a previous experiment, which compared ERP responses to highly expected words that were placed either in a MWE or a regular non-fixed compositional context, and to low predictability controls. We focused on oscillatory dynamics and regression analyses, in order to dissociate between the two contexts by modeling the electrophysiological response as a function of item-level parameters. A significant interaction between word position and condition was found in the regression model for power in a theta range (~7–9 Hz), providing evidence for the presence of qualitative differences between conditions. Power levels within this band were lower for MWE than compositional contexts when the target word appeared later on in the sentence, confirming that in the former lexical retrieval would have taken place before word onset. On the other hand, gamma-power (~50–70 Hz) was also modulated by predictability of the item in all conditions, which is interpreted as an index of a similar “matching” sub-step for both types of contexts, binding an expected representation and the external input.

## 1. Introduction

Using previous contextual information in order to anticipate the near future is a pervasive mechanism of human cognition (Bar, 2007), allowing for a fast response to complex stimuli. Such a top-down modulation of perception is also an essential part of language comprehension, where real-time disambiguation involves anticipations about most likely completions. Behavioral studies show that reading times for predictable words are shorter than for unpredictable ones (Ehrlich and Rayner, 1981), demonstrating how prior linguistic context can facilitate linguistic processing. Such predictions may be based on different types of information and occur at different levels. Prior semantic and syntactic content may be used to anticipate a concept and word class that may map onto several lexical items. On the other hand, within certain fixed expressions, a unique word may be unequivocally anticipated, leading to qualitatively different processing.

Previous studies addressing this issue (e.g., Molinaro et al., 2013) have been able to identify differences between compositional contexts and fixed expressions in the event-related potential response (ERP), however, whether this reflects a qualitative difference between the two, or just a stronger expectation in the case of fixed strings remains an open question. The present study aims to address this issue using item-level variability along a number of lexical and orthographic dimensions. Incorporating such item-level variables into the analysis of the electrophysiological response to each type of context will allow a better characterization of the underlying cognitive processes, thus informing neurophysiological models of sentence comprehension.

Within the ERP methodology, the N400 effect is tightly linked with predictability. This ERP component, initially described by Kutas and Hillyard (1980) as a response to semantically anomalous sentence endings, consists of an increased negativity peaking around 400 ms, with a broad scalp distribution. Its amplitude has since then been shown to correlate positively with the predictability of a target word as estimated by its Cloze Probability, (CP^1^: Kutas and Hillyard, 1984), by its word position in the sentence (Van Petten and Kutas, 1990), and through word probabilities derived from corpus-based models (Frank et al., 2013).

However, the functional interpretation of the N400 component has been debated (e.g., Molinaro et al., 2010). Firstly, an alternative account attributes its modulation not to predictability itself, but to ease of semantic integration (Brown and Hagoort, 1993). Under the predictability view, lexical retrieval would be facilitated through the contextual pre-activation of the given item, whilst under the integration view, facilitation would occur at a combinatorial processing stage, after recognition of the target word had taken place. Federmeier (2007) argues for the predictability interpretation using evidence from previous studies, such as Federmeier and Kutas (1999), that compared the N400 response to unlikely items that had different degrees of semantic similarity to the expected response but would pose similar integration demands (e.g., “They wanted to make the hotel look more like a tropical resort. So, along the driveway, they planted rows of *palms / pines / tulips*). The N400 response was larger for “*tulips*” than for “*pines*,” suggesting that anticipatory activation of “*palms*” would have led to a stronger concurrent activation of “*pines*,” given their shared semantic features (see also Rommers et al., 2013b for a similar paradigm, where anomalous target words sharing only shape-related features with the expected completion also elicited an attenuated N400 response).

Lau et al. (2008) reviewed available evidence from fMRI and MEG localization experiments that employed the same paradigms used in the N400 literature, finding that the only brain region that consistently shows effects under all the reviewed experimental settings is the posterior middle temporal cortex. This is taken as further evidence for the predictive account, given that this area is thought to be involved in lexical/conceptual retrieval, whereas ease or difficulty of integration with prior context should elicit effects in the anterior temporal, inferior parietal and inferior frontal regions.

Semantic constraints may thus facilitate processing at the lexical/conceptual retrieval stage, encompassing, however, a semantic field rather than a specific lexical item. In addition, some studies have been able to show earlier anticipatory effects, acting at the orthographic recognition stage. Kim and Lai (2012) compared the ERP response to semantically constraining sentences where a target word was replaced by an orthographically similar, or dissimilar, pseudoword (e.g., “She measured the flour so she could bake a *cake/ ceke / tont*…”), finding that relative to the expected item (*cake*), the similar pseudoword (*ceke*) elicited a positive deflection at 130 ms, whereas the dissimilar (*tont*) differed from the control, with a different pattern and at a later stage (enhanced negativity at 170 ms). They interpret such an enhanced detection of small, as compared to large, deviations from the target within an interactive top-down/bottom-up framework: when very early bottom-up analysis of the stimulus confirms the top down expectations generated at the conceptual level, further visual analysis stages are enhanced by a specific orthographic prediction. Such an account, whereby conceptual-level expectations percolate down to more specific, visual ones, has also been described at the neural level (Dikker and Pylkkänen, 2013). In an MEG picture-to-word priming task, before the noun was presented, the pictorial contexts elicited activation in left mid-temporal cortex (linked to lexical access), prefrontal cortex (associated with top-down processing) and visual cortex successively.

Nevertheless, early orthographic effects (related to ERP components earlier than the N400) are not as ubiquitous as semantic ones (reflected in the N400). Indeed, anticipating a specific item would in most cases be a difficult task and could lead, overall, to more processing costs than benefits (Jackendoff, 2002). At a semantic level this issue can be resolved by the idea that the expectation, encompassing a set of semantic features, would lead to facilitation of the expected item, but also of its semantic associates. However, given the arbitrary relation between form and meaning in the language system (words such *ant* and *mosquito* are semantically related but not form related), such a semantic neighborhood would not map onto an orthographic one, and pre-activation of the visual features of one word would be of no benefit when processing conceptually similar items. As Kim and Lai's study suggests, only when an initial visual analysis is highly congruent with the orthographic form of the expected item would perceptual top-down facilitation come into place, thus leading to a faster identification of orthographic anomalies.

However, predictions about linguistic stimuli may not be grounded on semantics alone. Associative relationships between words may determine that a specific lexical item, and no other, will appear: such is the case of multi-word expressions (MWE), where particular combinations of lexical items “crystallize” in our semantic memory (Cacciari and Tabossi, 1988, Tremblay et al., 2011). These expressions are pervasive in language, ranging from non-compositional idioms such as “*kick the bucket*,” where the meaning cannot be inferred from the sum of its parts, to collocations, where despite their compositionality, the specific units co-occur with a markedly high frequency, and in a fixed order (such as “*as good as gold*,” or binomials like “*knife and fork*” but not “*fork and knife*:” Siyanova-Chanturia et al., 2011, Arcara et al., 2012).

The ERP correlates to the comprehension of such expressions have been studied by several authors. Roehm et al. (2007a) employed antonym pairs as stimuli, where the second element in the pair was substituted by a same-category or unrelated violation (e.g., “*The opposite of black is white/yellow/nice*”), whilst Vespignani et al. (2010) and Molinaro and Carreiras (2010) used similar paradigms, where MWEs in Italian and Spanish respectively were embedded in sentences where the last item was replaced by a close synonym or a violation^2^. The results of both studies revealed significant graded effects on the N400 amplitude (violation > related item > expected item), but the ERP waveform for the expected completion displayed a particular morphology, with a positive deflection within the initial N400 time-range and a more posterior topography. The authors interpret this as an overlapping P3 response, reflecting the co-occurrence of two qualitatively different processes: a semantic-level anticipation (indexed by the N400), and a partially overlapping categorical target identification mechanism (indexed by the P3). Indeed, the P3b component, a positive deflection peaking around 300 ms with parietal scalp topography, is commonly associated with context updating. In the framework proposed by Kok (2001) it reflects a template-matching process, where an encountered stimulus is compared with an internal representation in a categorical identification process (is it a target or not).

One question that follows from the above studies is whether the hypothesized P3 component arises from the presence of associative relationships between words *per se*, or from the confirmation of a strong expectation that could also be generated by regular compositional contexts. The experimental manipulations consisted of a target-word that was highly expected in one condition (MWE), but unexpected in the others (substitution or violation), so that it is not possible to discern if it was the nature of the expectation or its strength that elicited the results observed. In order to address this question, Molinaro et al. (2013) compared target words that were either embedded in a MWE or in a highly constraining compositional context. By controlling for CP in both conditions, they were able to directly contrast the nature of the predictions: based on associative relationships in one case, and on semantic compositional constraints in the other.

Their results resembled those in previous studies, showing a distinct posterior scalp topography during the first part of the N400 time window (250–350 ms) in the case of MWE, as well as an increased positivity during this same interval that disappeared later on (400–500 ms). The authors interpret these results as support for the presence of two qualitatively different anticipatory processes: a categorical expectation about a specific lexical item (that may either be fulfilled or not), and a graded, semantic expectation (that could be fulfilled to a certain degree). The first process would be more prominent for MWE and the second for highly constraining semantic contexts.

Despite the above experimental results, ERP analysis alone cannot provide conclusive evidence regarding the existence of two qualitatively different cognitive processes during an N400-time window. Firstly, the EEG signal measured at scalp electrodes consists of activity generated by different neuronal populations: if two different sources or networks are active during overlapping intervals, only their summed activity will be recorded at the scalp. Secondly, the ERP averaging process leads to the loss of two kinds of information: (a) any kind of electro-physiological response that despite being time-locked to the stimulus has varying phase across trial (it will be therefore be canceled out through the averaging process); (b) how the effect of interest is modulated by the different lexical and sentence properties of single items.

The Molinaro et al. (2013) study attempted to address some of these limitations by complementing their ERP analysis with oscillatory analysis of EEG phase-locking values (PLV), a method that statistically measures the transient phase coupling between two brain signals in specific frequency bands. Before reading the target word, increased theta phase synchronization was found for the collocational context (over frontal-occipital channels). Furthermore, a positive correlation was found between the increased theta synchronization (before TW onset) and an early post-TW ERP effect (~120 ms) for the collocational condition only, suggesting that long-range interactions in the theta band support early visual-orthographic analysis of the TW in the case of collocations. However, such PLV results in a pre-TW interval cannot be used to dissociate between the hypothesized P300/N400 overlap.

The present study aims to complement this approach by using regression analysis of the time-frequency decomposition of the data collected by Molinaro et al. (2013) over an N400-like time window. The time-frequency decomposition will provide further information regarding the full dynamics of the EEG response to the stimulus (Makeig et al., 2004), by characterizing the amplitude of oscillations at different frequency bands. The regression analysis will allow the evaluation of whether the frequency characteristics during the time-window of interest (P300/N400 window: 200–600 ms) are influenced by different lexical variables under each condition. Form-based related characteristics, such as the number of orthographic neighbors, may affect the cost of stimulus evaluation and the difficulty of the target-identification task, thus modulating MWE processing (the P3 component is sensitive to both: Herrmann and Knight, 2001). In contrast, lexical and context-related characteristics (such as frequency of use or CP) might be more influential for compositional contexts.

In addition to providing a better characterization of the EEG signal, evidence from the time-frequency domain also has direct functional significance. Increases or decreases in power at certain frequency bands may reflect the dynamic coupling between different brain areas through synchronization of oscillatory activity, thus giving valuable information as to which functional networks become active at different processing stages. Within the language domain, general increases in gamma (>30 Hz) and theta (4–7 Hz) bands, and decreases in alpha (8–12 Hz) ranges have been described in the course of sentence comprehension, with different functional interpretations relating both to predictability and semantic processing (for a review, see Bastiaansen et al., 2012).

Power increases within fast oscillatory activity (gamma-band) can be interpreted as a coupling of near-by neuronal populations arising from successful predictive processing, where representations generated through top-down mechanisms are found to match those generated through bottom-up analysis of the stimulus. Such is the account that Wang et al. (2012) propose for their findings in a study comparing sentences where a target word had either a high CP, low CP, or constituted a semantic violation. They report a parametric modulation of the N400 response (high CP < low CP < semantic violation), but an increase in lower gamma-band power (40–50 Hz; from 0.2 to 1 s post-stimulus onset) over left and posterior electrodes only for the high CP condition. Rommers et al. (2013a) also report increases in gamma power for predictable words in compositional contexts as compared to semantically-related or unrelated violations, albeit over a higher gamma range (50–70 Hz). Interestingly, they also applied the same manipulation to idiomatic contexts, but in this case, no differences in gamma power were found across conditions. Furthermore, a direct comparison between correct compositional and idiomatic expressions revealed higher gamma power for the former in a 60–70 Hz range. They interpret these findings as evidence for the relative “switching off” of semantic operations during idiom comprehension.

Conversely, in a non-sentential paradigm, Dikker and Pylkkänen (2013) found that predictability effects concentrated on the theta band (4–7 Hz), both before and after target word presentation. They generated predictable or unpredictable contexts for single words using preceding pictures (e.g., picture of an apple vs. picture of a bag with several fruits followed by the word “*apple*”), and examined also the effect of a match or violation for the predictable condition. Before presentation of the target word, more theta band activity for the predictable contexts over left mid-temporal cortex is interpreted as an index lexical retrieval. After target word onset, the contrast between a match or mismatch of the expectation also showed effects in the theta band.

Indeed, results from sentential paradigms also show theta band power increases may accompany lexical retrieval, as well as semantic violations. Bastiaansen et al. (2012) suggest that theta band power increases during lexical retrieval may reflect the binding of semantic properties across distributed representations: the topography of theta-band power accompanying content words was found to be modulated by the semantic properties of the words being processed, so that items with auditory semantic properties elicited theta increases in areas overlying auditory cortex, whilst those with visual semantic properties did so in areas overlying occipital lobes. On the other hand, theta power increases as a result of semantic violations (Davidson and Indefrey, 2007, Wang et al., 2012) could reflect error detection processes.

A complementary view (Klimesch, 1999), attributes theta increases to the encoding of new information, whilst search and retrieval in long-term memory would involve de-synchronization in upper alpha band (~11–12 Hz), which positively correlated with memory performance. Klimesch related lower alpha band power (~8–10 Hz) to attentional processes, although the specific boundaries between theta and alpha sub-bands would be subject to high individual variability.

Outside the language domain, Karakaş et al. (2000) studied the decomposition of the P3 ERP component under different paradigms, finding that although it could be explained in terms of superposition of oscillations in lower frequency ranges (delta – 1–3 Hz and theta – 4–7 Hz), a larger amount of variance was explained by delta band oscillations at Pz, with power in this range correlating positively with P3 amplitude. As a result, the delta response is interpreted by the authors as reflecting matching and decision-making operations. Furthermore, Roehm et al. (2007b) re-analyzed the EEG results from the earlier-described antonym study (Roehm et al., 2007a), in order to further dissociate the hypothesized N400/P3 overlapping processes. Indeed, their results showed that the graded N400 response was reflected in qualitative differences in the frequency domain: a delta response (1–3 Hz), maximal at Pz was observed in a comprehension task for the expected antonym pairs only (both in total power and in the time-frequency decomposition of the ERP waveform), but no differences in this range were observed between the two violation conditions. In contrast, a response in the lower theta band (3.5–5 Hz) was reported for the unrelated violation only (although such an increase was not observed in total power, only in the frequency decomposition of the ERP waveform).

Based on the literature reviewed, we could draw the following hypotheses for the present analysis: First, if a categorical, target-identification mechanism is in place during processing of MWE (Roehm et al., 2007a, Molinaro and Carreiras, 2010, Vespignani et al., 2010), a P3-related increase in delta power during the N400 time-window (Karakaş et al., 2000, Roehm et al., 2007b) would be expected for MWEs relative to compositional contexts. A first, low-frequency analysis will therefore focus on the two high CP conditions only. Second, if gamma power increases reflect semantic operations in high predictability contexts (Rommers et al., 2013a), increases in such a power range from 200 ms onwards would be expected when expectations are semantically-based (compositional contexts as compared to low cloze probability controls, Wang et al., 2012), but not when they are based on associative relationships (MWEs as compared to controls), involving a visual, rather than a semantic expectation (Rommers et al., 2013a). However, the specific frequency bands where effects may be detected could be influenced by specific experimental settings and analysis methodologies, so that the whole frequency spectrum will be examined. Finally, if qualitative differences between associative and semantically-based anticipations exist, detected effects could be differently modulated by item-level parameters. Form-based characteristics might be influential for associative anticipations (modulating the difficulty of the target-identification mechanism, Herrmann and Knight, 2001) whilst meaning-based factors could modulate the semantically-based predictions.

## 2. Materials and methods

### 2.1. Participants

Thirty-six right-handed native Spanish speakers took part in the experiment (mean age: 22.9, *SD* age: 5.2; 31 females), receiving €10 in exchange for their collaboration. They were all right-handed and had no history of neurological disease. Their vision was normal or corrected to normal.

### 2.2. Materials

A set of 88 target words (TW) were embedded in three kinds of sentences: collocational contexts, where the TW was the last item in a multi-word expression (MWE)^3^; semantically high-constraining contexts (SEM), where the TW was highly predictable, but not part of a fixed string; and semantically low-constraining sentences (CTR), where the TWs were unpredictable given their previous context, but nevertheless congruent. Target words were the same, and located in the same position within the sentence across conditions at the item level. They were never the last item of the sentence and were always content words. Their cloze-probabilities (as evaluated by an independent group of 40 native Spanish speakers) were very high for the MWE and SEM and did not statistically differ amongst themselves (MWE: Mean: 82.42, *SE*: 2.56; SEM: Mean: 81.56, *SE*: 2.08; *t*_(87)_ = 0.27); CP of TW in the control (CTR) condition was zero.

The MWE used in the first condition were more than three words long (Mean: 4.05, *SE*: 0.10). They were also very frequent expressions, as demonstrated by their frequency of occurrence (Mean: 829.51, *SE*: 215.11) in the *Corpus de Referencia del Español Actual* (http://corpus.rae.es/creanet.html), and highly familiar, as evaluated through a questionnaire given to 54 independent native Spanish speakers (mean rating: 5.87, *SE*: 0.19, on a 7 point scale where 1: never heard; 7:heard very often). Lexical characteristics (frequency, orthographic neighbors, and length) of the word preceding the target were also controlled for (no *t*-value larger than 1.32), which was often a function word (CTR: 53; SEM: 48; MWE: 52), and in the remaining cases a content word. This assured that no differences between conditions in the pre-target word interval could derive from the lexical properties of the preceding word, thus minimizing possible uncontrolled carry-over effects. For further details regarding the materials, see Molinaro et al. (2013).

The final experimental set of stimuli was comprised of 264 sentences (see Table 1 for examples), and an additional 12 sentences used in a practice session.

**Table 1:** **Examples of sentence stimuli**.

**Condition**	**Example**
MWE	Aunque todos éramos incrédulos al respecto, todo se solucionó como por arte de **magia** cuando más falta hacía.
	*Although we were all skeptical about the issue, everything was solved “as if by art of magic” when it was most needed*.
SEM	El mago nunca revela sus trucos, siempre dice que ha sido cosa de **magia** y no tiene explicación.
	*The magician never reveals his tricks, he always says it was just magic, and cannot be explained*.
Control	Como estábamos muy estresados Eneko y yo, acudimos anoche a un espectáculo de **magia** y de humor.
	*Since we were feeling very stressed, Eneko and I went to a magic and humor show last night*.

*Target word (TW) appears in bold. English translation for the multi-word expression (quoted values) is literal*.

### 2.3. Procedure

Participants were tested individually in an electrically-shielded room. Sentences were presented on a CRT computer screen one word at a time. Each word remained on screen for 300 ms and was followed by a 300 ms blank. Yes/No comprehension questions were presented every five sentences on average and sentence order was fully randomized. Twelve practice trials were provided before the experimental session started, which lasted 1 h and 15 min including five breaks across the session. EEG data was simultaneously recorded using BrainAmp system (Brain Products GmbH), through 32 electrodes, at a sampling rate of 500 Hz. Twenty-seven of these were mounted on an EasyCap according to the 10–10 international system (Fp1/2, F3/4, F7/8, Fz, FC1/2, FC5/6, C3/4, Cz, T7/8, CP1/2, CP5/6, P3/4, Pz, P7/8, O1/2), with two electrodes placed on the two mastoid bones and an additional four facial electrodes (two electrodes placed below the two eyes and two electrodes placed on the external chanti of both eyes). Recording was on-line referenced to the left mastoid. Scalp and mastoid electrode impedance was kept below 5 kOhm, and below 10 KOhm for the horizontal eye positions. For further details regarding the procedure, see Molinaro et al. (2013).

### 2.4. Time-frequency analysis

Data analysis was carried out in Matlab 2010b, using the FieldTrip toolbox (Oostenveld et al., 2011). EEG was re-referenced offline to the average activity of the two mastoids and filtered with a 0.1–120 Hz band pass filter. The recordings were segmented in time intervals between −1800 and 1000 ms relative to the presentation of the target word. Eye movements, blinks and electrocardiographic artifacts were reduced using independent component analysis (Jung et al., 2000), with subsequent visual inspection of the data to remove any epochs with remaining artifacts. Data from two participants were discarded due to rejection of a high number of trials, and of a further participant due to accidental loss of codes indicating order of trial presentation. From the remaining 33 participants, 6.1% of trials were rejected on average, with no significant across-condition differences [*F*_(2, 66)_ = 1.11, *p* = 0.3].

EEG data were then demeaned to eliminate channel bias, by subtracting the mean over the entire epoch from each amplitude value. The time-varying power spectrum of single trials was obtained using two different techniques: a multi-taper approach (Mitra and Pesaran, 1999) for the gamma-range (30–80 Hz) and a Hanning window (500 ms window, 2 Hz frequency steps, 40 ms time steps) for the lower frequencies (0–30 Hz). In the multi-taper analysis, power was calculated using three orthogonal tapers and a time-varying taper length for each frequency (fitting 5 cycles), so that the temporal smoothing decreased with higher frequencies. Time and frequency steps of the sliding window were the same as for the Hanning analysis. Power values were expressed as relative change from a baseline interval calculated from −950 - −650 ms. This is a 300 ms interval prior to the presentation of the word preceding the target (TW-1), rather than the TW itself, which allows direct comparison with the ERP results presented by Molinaro et al. (2013), and minimizes the presence in the baseline of any pre-stimulus predictability effects.

### 2.5. Statistical analysis

#### 2.5.1. Confirmatory analysis

Statistical comparisons (for each time, frequency, and electrode over the hypothesized windows) were performed through non-parametric permutation-based *t*-tests (MWE vs. SEM comparison) and *F*-tests (involving all three conditions), using 1000 permutations. We hypothesized differences between the two high-expectancy conditions in the delta band, so the two-way comparison (MWE vs. SEM) was used for a low frequency range (1–3 Hz) over an N400-like time window (200–600 ms, Kutas and Federmeier, 2011). On the other hand, we expected differences in the gamma band between low and both types of high CP items, so all conditions were contrasted for a high frequency range (40–70 Hz) encompassing the one described by Wang et al. (2012) and Rommers et al. (2013a).

#### 2.5.2. Exploratory analysis

The above analysis was then extended to include the full frequency range (0–70 Hz), in order to identify other effects not predicted by our hypotheses.

In addition, these comparisons (both for the confirmatory and exploratory analyses) allowed us to further specify the time (ms), frequency (Hz) and space (electrodes) intervals to be considered in the following mixed-effects analysis. Such a selective analysis avoids circularity (Kriegeskorte et al., 2009) by using independent criteria for data selection (differences in the means across conditions) and statistical inference (correlation between power values and several item-level variables). The only predictor in the models that would suffer from circularity is *condition*. Since our selection procedure was based upon differences in condition means, no statistical inferences can be drawn from the presence of a main effect of *condition* in the regression models.

#### 2.5.3. Mixed-effects multiple regression

The log-transformed power averaged over the selected windows served as the dependent variable against which a mixed-effects multiple regression analysis with crossed random effects for subjects and items (Baayen et al., 2008) was performed.

Several item-level variables covering both form-based and meaning-based characteristics of the TWs were included as independent variables in the models (see Table 2 for descriptive statistics):

- *Number of characters (NRCHAR):* A number of low-level lexical factors, such as number of characters, font type and size, have been reported to affect reading times (Rayner and Pollatsek, 1987). With regard to ERP components, word length affects early stages of processing (~100 ms), probably reflecting visual analysis of the stimulus, without interacting with the semantic processing of the item (Hauk et al., 2006). Since monospaced fonts were used in the experiment, physical word length could be measured by the number of characters.
- *Orthographic neighbors (NEIGHB):* As with word length, the number of orthographic neighbors (visually similar items, such as *“cat”/“car”*) affects orthographic discrimination of words and can influence both RTs (e.g., McClelland and Rumelhart, 1981) and ERPs (Holcomb et al., 2002). These values, estimated as the Levenshtein distance, were obtained from the *EsPal* database (Duchon et al., 2013).
- *Single word frequency (LOGFREQ):* The effects of word frequency in reading have been repeatedly reported (e.g., Juhasz and Rayner, 2003), although the degree to which this reflects a form-based or meaning-based facilitation derived from familiarity can be questioned (familiarity with the written word-form vs. familiarity with the concept). Baayen (2005) suggests that the tighter correlation of this measure with other word meaning, rather than word form measures, indicates that word frequency mainly indexes conceptual familiarity. Log-transformed word-frequency estimates were obtained from the EsPal database (Duchon et al., 2013).
- *Word bigram frequency (LOGFREQBI):* The frequency of occurrence of two word sequences has also been shown to affect reading times (for a review, see Tremblay, 2012). Log-transformed bigram frequency estimates calculated from bigram counts (CREA corpus) were included in the models in order to control for such effects.
- *Cloze Probability (CLOZEPROB):* The main object of this study is to explore whether the differences in predictive processing of highly predictive compositional vs. associative contexts are qualitative or quantitative. As such, including in the model a measure of predictability allows the estimation of the effects of condition, once quantitative differences in predictability are accounted for. Although cloze probability for our conditions of interest was always high, there was enough variability to allow its inclusion as continuous predictor of power (see Table 3). In addition, its values were log transformed to obtain a better spread.
- *Word position (WORDPOS):* Word position in a sentence has been shown to influence RTs, N400 amplitude (Van Petten and Kutas, 1990), and also power estimates over certain frequency bands (Bastiaansen et al., 2002). This has typically been interpreted as a predictability effect: as a sentence develops, higher semantic constraints are placed on upcoming items. This variable was codified as position of the target word from the beginning of the sentence.
- *Trial number (TNUMBER):* Sentence position in the experimental list was included in order to control for fatigue or practice effects.

**Table 2:** **Item-level variable descriptive statistics**.

**Variable**	**Condition**	**Range**	**Median**	**Mean**	***SD***
Wordpos	Both	8–24	17.00	17.39	3.23
Nrchar	Both	3–11	5.00	5.45	1.62
Neighbors	Both	1.00–2.60	1.50	1.45	0.39
Logfreq	Both	0.42–3.14	2.15	2.00	0.66
Logfreqbi	MWE	0.09–2.27	0.72	0.89	0.64
	SEM	0.00–2.26	0.78	0.57	0.76
CP	MWE	10–100	92.99	82.22	24.08
	SEM	40–100	90.00	81.56	19.55

**Table 3:** **Selected windows for mixed-effects analyses**.

**Window**	**Time**	**Frequency**	**Channels**
1. Theta/delta	400–600 ms	2–4 Hz	CP1, CP2, P3, Pz, P4
2. Alpha/theta	260–420 ms	7–9 Hz	F7, F3, FC5, T7
3. Gamma	220–300 ms	50–70 Hz	FC5, T7, CP5, FC1, C3, CP1

Initial models included by-subject and by-item intercepts as random effects, and as fixed effects all the item-level variables (centered and scaled) in addition to the interaction of each with a categorical *condition* factor (SEM = 0; MWE = 1). Final models were built by back-fitting fixed effects and forward fitting by-subject and by-item random slopes. First, predictors with |*t*| < 2 were removed one at a time, starting with the interaction terms. The significance of each predictor was assessed through log-likelihood tests, so that only those that improved model fit (*p* < 0.05) were kept in the models. By-subject and by-item random slopes were then assessed individually using likelihood tests.

Outlier removal was handled after model fitting, since mixed-effect modeling is less vulnerable to extreme values that can critically affect other analyses highly dependent on means aggregation (Baayen and Milin, 2010).

## 3. Results

### 3.1. Windows of interest

Statistical comparisons of the spectral-power estimates were performed using the Resampling Statistical Toolkit, part of the EEGLAB toolbox Delorme and Makeig (2004) for Matlab. The obtained *p*-values were corrected through the false discovery rate (FDR) method (Benjamini and Yekutieli, 2001), but under this correction, conservative with small effects, no significant differences were found for any of the contrasts in the confirmatory or exploratory analyses. No strong differences between conditions could therefore be detected using averaging-based analysis techniques.

Since the focus of the present study is to use item-level properties to characterize the frequency response in each condition, windows of interest were identified using uncorrected *p*-values (set at an α = 0.01), and subjected to a certain degree of smoothing through inspection of *t*- and *F*-maps masked with a more liberal threshold (0.05).

#### 3.1.1. MWE vs. SEM contrast, low frequency bands (0–30 Hz, 0–600 ms post TW)

**Figure 1:**
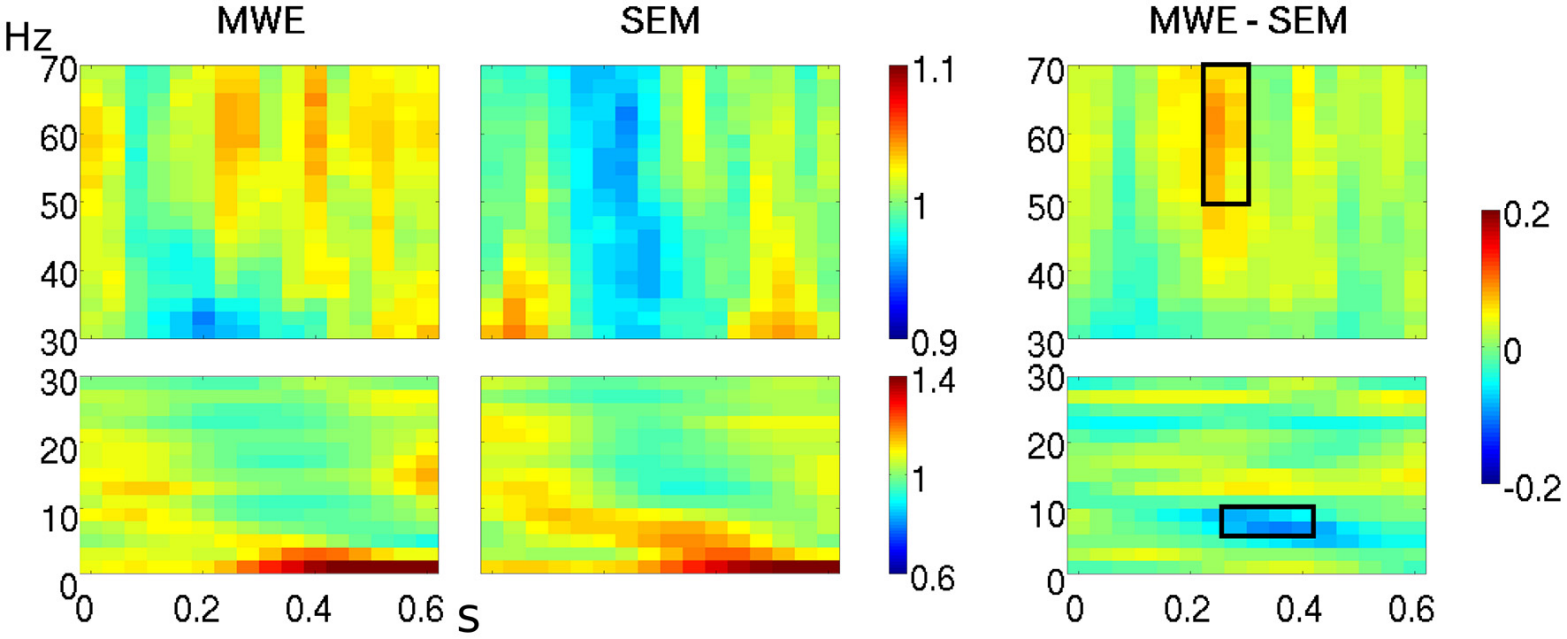
**Time-frequency representations of the two high expectancy conditions (MWE, SEM) at electrode T7**. High and low frequency ranges are represented separately. The third panel shows the contrast between both conditions, with the selected window for further analysis outlined in black.

**Figure 2:**
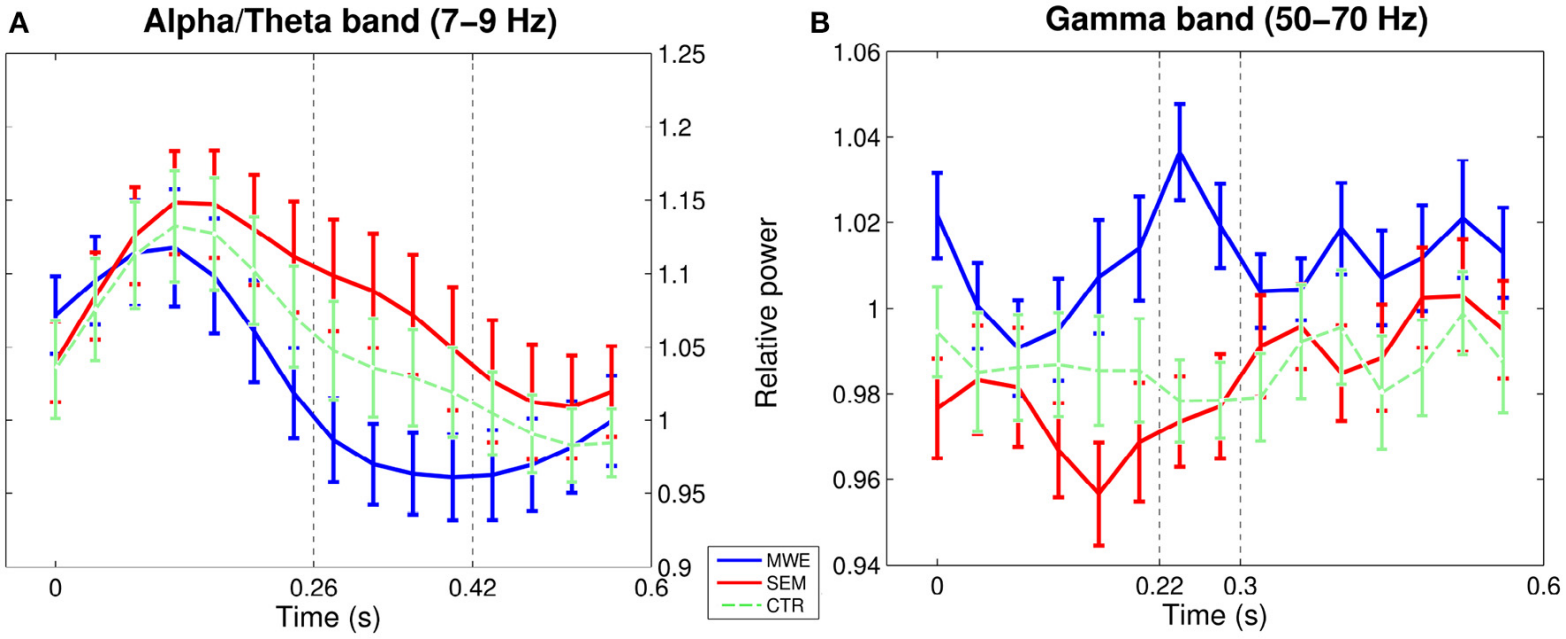
**Temporal evolution of power between 0 and 0.6 s post TW onset over selected channel-frequency windows: (A) alpha/theta, (B) mid gamma band**. Error bars indicate standard errors around the mean, for illustrative purposes; dotted lines mark analyzed time-window. Although the control condition was not analyzed in the low frequency contrast, it is included in the figure as a reference.

The *t*-maps (*p* < 0.01, uncorrected) showed two windows which were selected for further analysis (see Table 3):

1. At the boundary between delta and theta bands (2–4 Hz), from 400–600 ms over parietal electrodes (CP1, CP2, P3, Pz, P4). Power over the selected interval was lower for MWE (mean: 1.06, *SE*: 0.03) as compared to SEM (mean: 1.12, *SE*: 0.03).
2. At the boundary between alpha and theta bands (7–9 Hz), from 260–420 ms over left frontal and temporal electrodes (F7, F3, FC5, T7). Power over the selected interval (see Figures 1, 2) was lower for MWE (mean: 0.97; *SE*: 0.03) than SEM (mean: 1.08; *SE*: 0.04).

#### 3.1.2. All conditions analysis, high frequency bands (30–70 Hz, 0–600 ms post-TW)

The one-way ANOVA *F*-maps (contrasting the three conditions MWE, SEM, and CTR) showed differences within an upper gamma band window (50–70 Hz) in the 220–300 ms interval, over left lateralized electrodes (FC5, T7, CP5, FC1, C3, CP1). Figures 1, 2 show that power within this frequency during this time-interval is higher for MWE (mean: 1.04, *SE*: 0.01) than SEM (mean: 0.97, *SE* = 0.01), with CTR showing an intermediate pattern (mean: 0.98, *SE* = 0.01).

### 3.2. Mixed-effects models

Data was analyzed using the free software statistical package R (R Core Team, 2013) and the *lme4* and *lmerConvenienceFunctions* libraries (Tremblay, 2011; Bates et al., 2012 respectively). Correlations amongst some of the predictors were high, especially between orthographic neighbors and number of characters [*r* = 0.77, *t*_(86)_ = 10.18, *p* < 0.001]. However, multicollinearity diagnostics showed that the problem was not severe (a kappa test on the baseline predictors gave a condition value, κ, of 6.94, indicative of mild co-linearity).

#### 3.2.1. Window 1: delta/theta (2–4 Hz)

Neither of the single-item predictors nor their interactions with *condition* were found to be significant.

#### 3.2.2. Window 2: theta/alpha (7–9 Hz)

A *condition* by *word-position* interaction was found to be significant by a likelihood test comparing the model with and without the interaction (χ^2^_(1)_ = 3.83, *p* = 0.05; see Table 4 for model coefficients). No by-subject or by-item slopes were significant. Exploration of quartile-quartile plots and residuals revealed normality and homoscedasticity, indicating that the model was coping well with the data, so no further outlier removal was performed. Variance for the random effects was 0 for by-item intercepts and 0.008 for the by-subject intercepts, with a residual variance of 1.24. Following Barr et al. (2013), we also built a maximal model including by-subject slope for the *condition-by-word-position* interaction (the model did not converge when also including a by-item slope). The fixed effect estimate for the *condition by word position* interaction did not differ from the results reported in Table 4, although there was a slight drop in the corresponding *t*-value (−1.92), and in the χ^2^ statistic from the likelihood ratio test (χ^2^_(1)_ = 3.65, *p* = 0.056).

**Table 4:** **Fixed effects for Theta/Alpha models**.

**Model**	**Fixed effects**	**Estimate**	***SE***	***t*-value**
MWE coded as 1	(Intercept)	0.230	0.026	8.58
	Wordpos	0.025	0.021	1.16
	Cond	−0.029	0.030	−0.94
	Cond:wordpos	−0.059	0.030	−1.96
SEM coded as 1	(Intercept)	0.197	0.026	7.53
	Wordpos	−0.035	0.021	−1.61

*Values for condition and condition-by-word position interaction for both models are the same, and therefore not reported for the second model*.

The *condition* by *word-position* interaction was tested by fitting an additional model where the *condition* factor was re-coded (MWE = 0, SEM = 1), so that the coefficient for word position reflects the simple slope for each group. The correlation between power and word position was positive for SEM, and negative for MWE, being stronger for the latter (see Table 4).

#### 3.2.3. Window 3: gamma (50–70 Hz)

For the gamma frequency range, *condition* and *cloze probability* remained as predictors in the final model (|*t*| > 2). The significance of *Cloze probability* was confirmed by a log-likelihood ratio test (χ^2^_(1)_ = 4.33, *p* = 0.04). Power levels were higher for the MWE than the SEM condition. Exploration of quartile-quartile plots and residuals revealed that the model was not coping very well with extreme values, deviating from normality. The data was therefore trimmed, eliminating data-points whose residuals were more than 3.5 *SD* away from the mean (29 data points were removed), resulting in a much better model fit (see Table 5 for trimmed model coefficients). Variance for the random effects was 0.0001 for by-item intercepts and 0.0003 for the by-subject intercepts, with a residual variance of 0.24. Estimates for *cloze probability* fixed effect remained the same after fitting a model with a maximal random effect structure, with χ^2^ values obtained through a log-likelihood ratio test dropping slightly (χ^2^_(1)_ = 3.97, *p* = 0.05).

**Table 5:** **Fixed effects for trimmed Gamma model**.

**Fixed effects**	**Estimate**	***SE***	***t*-value**
(Intercept)	−0.061	0.010	−6.07
Cond	0.048	0.014	3.57
Clozeprob	0.014	0.007	2.06

## 4. Discussion

The present study aimed to investigate whether different brain dynamics underlie the predictive response to words embedded either in regular compositional contexts or in MWEs. In the former case, prior semantic information would be used in order to anticipate an upcoming concept and the corresponding likely word candidate. This process, previously linked to the N400 component, would be graded and modulated by the conceptual similarity of the expected item to the actually encountered one. However, several authors (Roehm et al., 2007a, Molinaro and Carreiras, 2010, Vespignani et al., 2010) have proposed that under the associative contexts generated by fixed strings, a categorical expectation is generated, leading to prior lexical retrieval of the upcoming word.

In the case of multi-word expressions, the visual recognition process during reading would thus be akin to a target-identification mechanism, where the encountered stimuli would be compared to an internal representation. Such a process could be indexed by the presence of a P3 effect in comparison to regular compositional contexts. Molinaro et al. (2013) examined this question by comparing MWEs to highly constraining compositional contexts, finding evidence for the presence of qualitative differences between conditions, through a phase-locking value analysis that revealed differences before presentation of the target word, as well as through an event-related potentials analysis suggesting the presence of a P3 effect for fixed strings. However, the additive nature of the EEG signal and the averaging procedure of the ERP analysis do not allow for conclusive results in this regard. The present study aimed to find further evidence of qualitatively different processes in the post-stimulus interval using time-frequency decomposition of the EEG signal, and regression statistical analyses characterizing the frequency response in terms of item-level variables.

We expected to find differences in two frequency bands: in a delta range, during 200–400 ms and in a gamma range, from 200 ms onwards. Previous research had linked an increase in delta power to target identification mechanisms and the P3 component, during reading of fixed expressions (Roehm et al., 2007b) as compared to compositional contexts, but also in non-linguistic domains (Karakaş et al., 2000). In addition, Wang et al. (2012) reported increases in gamma power during reading of highly expected words as compared to low cloze probability controls, whereas Rommers et al. (2013a) showed that gamma power was higher for semantically constraining contexts than for idiomatic expressions. However, our results revealed no statistically significant differences when comparing power levels averaged over all trials for each condition over the hypothesized time-frequency windows, or over the whole spectrum after correcting for multiple comparisons.

On the one hand, *a priori* determination of frequency bands may miss effects present in the data: small differences between studies employing similar paradigms may lead to substantial differences in the frequency response (see discussions in Klimesch, 1999: regarding individual differences, and Davidson and Indefrey, 2007: regarding the impact of rate of presentation). On the other, statistical comparisons of the full time-frequency-channel data averaged over linguistically variable items may lack the power to detect small effects after correcting for multiple comparisons.

We therefore took an alternative strategy. We used a data-driven approach to select windows of interest (based on maximizing differences between time-frequency-channel data averaged over trials in each condition), and performed a regression analysis to assess how item-level properties modulated the power response (averaged over the selected time-frequency-channels) in each condition, focusing our statistical inference on the latter. This allowed us to evaluate whether both conditions differed in a qualitative way through the presence of condition-by-lexical variable interactions, even when we could not draw inferences regarding differences in the overall means due to the lack of significant results in the selective analysis. Furthermore, the presence of significant main effects of any of the item-level variables may provide information regarding the underlying cognitive processes indexed by power in the given range. In this way, one of the three windows identified (in delta frequency range) was discarded, as no predictors were significant in the mixed-effects model except for condition. We concentrate further discussion on the remaining windows.

### 4.1. Low frequency responses

**Figure 3:**
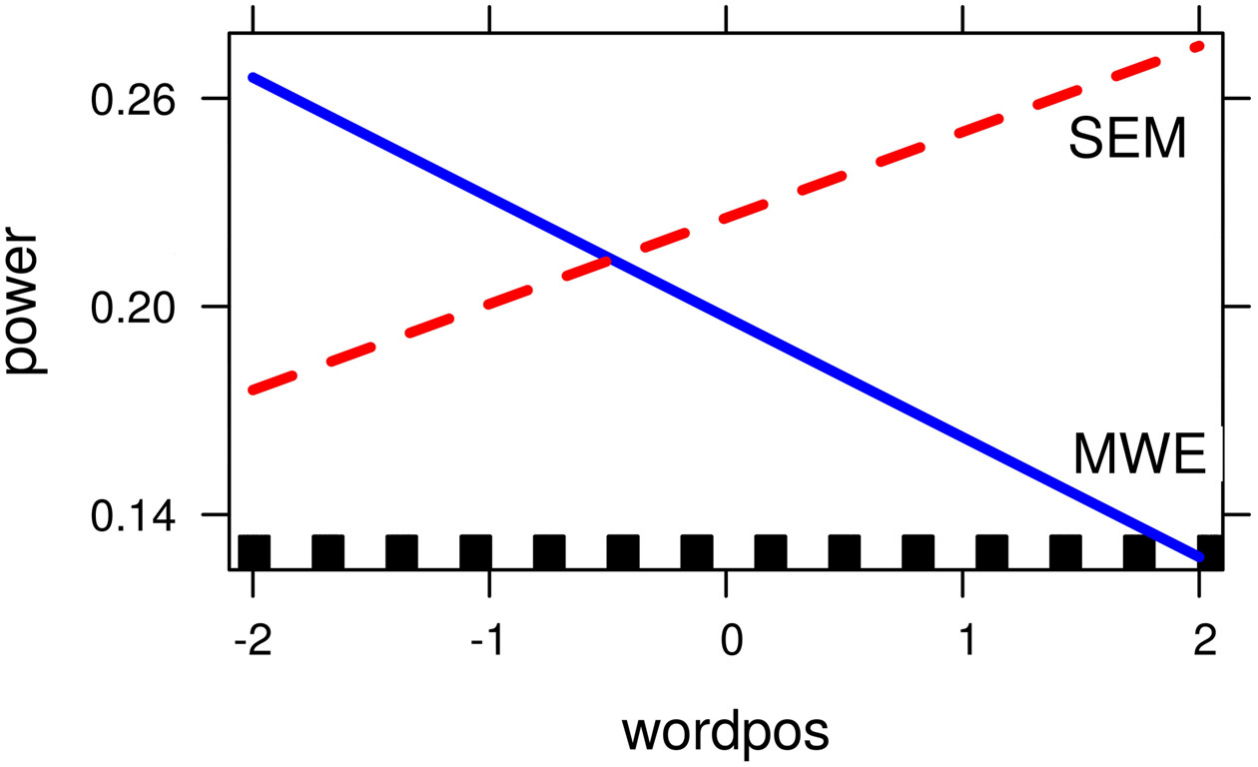
**Word-position by condition interaction for Alpha/Theta band model (6–9 Hz)**. Axis show transformed values for the dependent and independent variable: logarithm for the relative power values, and centered values for word position.

Following the two-way contrast between the semantically constraining sentences and those containing fixed expressions, a cluster at the theta/alpha boundary (6–9 Hz) from 260 to 420 ms over frontal and temporal electrodes in the left hemisphere was selected for further analysis. Overall mean power within this window was lower for MWEs than for compositional contexts (mean: 0.97; *SE*: 0.03 vs mean: 1.08; *SE*: 0.04). However, the regression analysis revealed a condition-by-word-position interaction showing that the differences in power between the two conditions were not constant across the sentence. Theta power was negatively correlated with word position only in the case of fixed strings, and seems to be lower than for compositional contexts only when the target word occurs later on in the sentence, where differences between conditions are maximal (see Figure 3).

Such a frequency range, between 6 and 9 Hz could be interpreted as a lower alpha or as a theta effect, given the high inter-individual variability in alpha band frequencies (Klimesch, 1999). Lower-alpha desynchronization has been linked to attentional processes, whilst theta-band synchronization has been linked to lexical-semantic retrieval (Bastiaansen and Hagoort, 2003). However, both the topography (left hemisphere) and the timing of the cluster are more consistent with the language-related theta effects described by Bastiaansen and Hagoort (2003).

Taking theta power to be an index of lexical retrieval, our hypotheses would predict lower power levels for MWEs than compositional contexts: In the case of MWEs only, retrieval of the whole lexical bundle would have taken place at an earlier time-point in the sentence, once the expression is recognized as such (recognition point, see Vespignani et al., 2010). In the case of semantically constraining sentences, an anticipatory facilitation could lead to a certain degree of pre-activation, but full retrieval would still require visual recognition of the upcoming item.

However, our results show that the differences in theta power between the two conditions is modulated by target word position, with the expected pattern (lower values for fixed strings) being strongest when the word appears later on in the sentence. If prior lexical retrieval at the recognition point is responsible for differences in theta-band synchronization, it follows that such a recognition point is dependent upon word position in the sentence. The absence of strong semantic constraints at the beginning of a sentence might delay the recognition point to the last element of a fixed expression, so that full retrieval of the lexical bundle would coincide with recognition of the target word. As the sentence unfolds, the increase in contextual semantic information (preceding the onset of the MWE) can lead to an earlier recognition of the fixed expression, allowing for full lexical retrieval of the fixed string before the target word is actually encountered.

We did not find evidence to support our first hypothesis, that predicted P3-related delta increases for fixed strings as compared to compositional contexts. This could be related to differences in the paradigms employed: Roehm et al. (2007b) compared the response to a highly expected antonym with a related-substitution that was nevertheless unexpected. In contrast, in the Molinaro et al. (2013) paradigm both conditions had a high cloze probability. In addition, Roehm et al. showed that the delta response was contingent on the task employed, and could not be detected when it involved lexical decision rather than comprehension. Although the paradigm used in the present study also involved a comprehension task, it differed with the one employed by Roehm et al. in another important aspect: the stimuli included only correct sentences, with no violations.

### 4.2. High frequency response

Following from the results reported by Wang et al. (2012) and Rommers et al. (2013a), we expected to find predictability-related increases in gamma (40–70 Hz) synchronization from 200 ms onwards (Wang et al.'s effect persists over 1 s) for the semantically constraining contexts as compared to controls and as compared to MWEs. However, our three-way comparison between all conditions revealed no significant differences after correcting for multiple comparisons.

Subsequent window-selection procedure identified a smaller time-window (~200–300 ms), for a gamma range between 50 and 70 Hz, that was further analyzed using mixed-effects models. Interestingly, the regression model provided evidence that gamma power within this range was indeed related to predictability, with cloze probability being a significant positive predictor of power. There was no significant interaction between this predictor and condition, showing that such a relationship held true across the two high predictability contexts. However, gamma power for the low cloze-probability controls was not lower than for the semantically constraining contexts (mean: 0.98, *SE* = 0.01; mean: 0.97, *SE* = 0.01, respectively). This discrepancy could be explained in terms of differences in the baseline interval used to calculate relative power values. Although the characteristics of words prior to the target were carefully controlled for in Molinaro et al. (2013), cloze probabilities of words preceding the target were considerably lower for controls than for the two high expectancy conditions (see Table 1 in Molinaro et al.). In addition, whether the positive relationship between cloze probability and power held true within the control sentences could not be assessed given the low variability of cloze probability in this condition. For this reason it is critical to evaluate relative differences between the two high expectancy contexts.

Our data is thus consistent with Wang et al.'s (2012) results linking gamma to predictability, but contrary to Rommers et al. (2013a), we cannot link this frequency range to semantically-based anticipations: gamma power was higher for words embedded in idiomatic experessions than for semantically-constraining contexts (see Figure 2). Such a discrepancy could be explained in terms of task differences: whilst Rommers et al. used a paradigm that included sentences with expectation violations, our experimental stimuli only contained correct sentences. The proportion of expectation violations in an experimental set has been shown to modulate the the N400 effect (Lau et al., 2013), and cognitive factors like attention Gruber et al. (1999) can modulate gamma-band activity. Attentional patterns may differ in each experimental setting: In a context where only correct sentences are seen an appropriate processing strategy would be to rely on top-down predictions regarding the upcoming word. On the contrary, within the presence of violations more attentional resources may be devoted to bottom-up analysis of the stimulus. If gamma power can be related to predictability across different levels of the cognitive hierarchy, attention-related task differences may modulate at which level (semantic or visual) predictability effects may be enhanced, and therefore detected.

Interestingly, the temporal evolution of power in our case also appears to be different to the one reported by previous studies. Whilst Rommers et al. report gamma synchronization post target word that persists over 1 s for the semantically constraining condition, our results show successive increases and decreases in power values for the two high predictability conditions during the first ~300 ms, that are nevertheless out of phase, resulting in maximal differences between conditions between 220 and 300 ms (interval that was detected by our data-selection analysis). In contrast, power levels for the control condition remain fairly stable during the whole post-target word interval.

A tentative explanation for such a pattern would be a gamma-rhythm modulation by theta-band oscillations, mechanism that has been proposed to integrate local cell assemblies into large-scale networks (for a review, see Buzsáki and Wang, 2012). Top-down modulation driving the activation of the expected representation would involve large-scale network synchronization in the theta band, whilst successful match with the encountered stimulus could lead to a local increase in gamma-synchronization. Through cross-frequency coupling of gamma power with the theta-rhythm, information about the success of the match may be incorporated into the large-scale network. This process would not be in place for our low predictability sentences, where a successful match is not expected. In addition, the differences in phase between power oscillations for the two high probability conditions could reflect differences in the timing of the predictability response, with an earlier confirmation of the expectation for the case of MWE. It is important to note, however, that this is only a tentative explanation based on visual inspection of the plots, pointing to an interesting avenue for further analysis of this data-set.

### 4.3. Final remarks

In sum, our results provide further evidence of a qualitative difference in anticipatory processing of fixed strings and regular compositional contexts, as evidenced by the differential influence of word position on power in a theta-like range for each type of context. Modeling the frequency response as a function of different item-level variables thus allowed us to better characterize the cognitive processes under each condition, even in the absence of statistically-detectable differences in the overall means.

We suggest that qualitatively different top-down modulation processes in a pre-TW interval could be leading to a pre-activation of certain lexical entries in the case of semantically constraining sentences, and to full retrieval for MWE. Upon encountering the target word, this would lead to subsequent facilitation in lexical retrieval in the former, and a decision to classify the stimulus as a target in the latter. However, the matching step between the bottom-up and the top-down generated representations (whether through full retrieval or pre-activation of an item) would involve the same gamma-band synchronization mechanism, which could show quantitative modulation: earlier in time and with a higher intensity for MWE than compositional contexts. However, our analysis followed an exploratory methodology, so that further research is needed in order to confirm the presented results.

In future studies, we intend to better characterize the different steps of these anticipatory mechanisms, by analyzing a pre-target word interval. It will be interesting to consider how lexical characteristics of the yet-to-come target word influence effects in this time period, and to quantitatively assess cross-frequency coupling. Using MEG and source reconstruction techniques together with individually-determined frequency bands may also enhance the power of the experimental set-up.

Finally, future research into the prevalence and importance of associative relationships between words may bring new insights to our understanding of language function and use. MWEs may play a special role in language, by providing “ready-made” strings to be directly retrieved from memory, thus relieving demands on working memory (Skehan, 1998, Bybee, 2006). The extent to which language relies on such strings, rather than pure compositionality, remains an open question.

### Conflict of interest statement

The authors declare that the research was conducted in the absence of any commercial or financial relationships that could be construed as a potential conflict of interest.

## Footnotes

^1^Percentage of subjects who complete a sentence fragment with a given word.

^2^The violation condition was only included in the Vespignani et al. study, whilst the Molinaro and Carreiras included additional manipulations.

^3^These sentences were selected from the stimuli used by Molinaro and Carreiras (2010), which included multi-word expressions extracted from the CESS-ECE corpus (Martí and Taulé, 2007).
